# LPI Radar Waveform Recognition Based on Time-Frequency Distribution

**DOI:** 10.3390/s16101682

**Published:** 2016-10-12

**Authors:** Ming Zhang, Lutao Liu, Ming Diao

**Affiliations:** College of Information and Telecommunication, Harbin Engineering University, Harbin 150001, China; zhangming@hrbeu.edu.cn (M.Z.); diaoming@hrbeu.edu.cn (M.D.)

**Keywords:** LPI radar, time-frequency distribution, digital image processing, waveform recognition

## Abstract

In this paper, an automatic radar waveform recognition system in a high noise environment is proposed. Signal waveform recognition techniques are widely applied in the field of cognitive radio, spectrum management and radar applications, etc. We devise a system to classify the modulating signals widely used in low probability of intercept (LPI) radar detection systems. The radar signals are divided into eight types of classifications, including linear frequency modulation (LFM), BPSK (Barker code modulation), Costas codes and polyphase codes (comprising Frank, P1, P2, P3 and P4). The classifier is Elman neural network (ENN), and it is a supervised classification based on features extracted from the system. Through the techniques of image filtering, image opening operation, skeleton extraction, principal component analysis (PCA), image binarization algorithm and Pseudo–Zernike moments, etc., the features are extracted from the Choi–Williams time-frequency distribution (CWD) image of the received data. In order to reduce the redundant features and simplify calculation, the features selection algorithm based on mutual information between classes and features vectors are applied. The superiority of the proposed classification system is demonstrated by the simulations and analysis. Simulation results show that the overall ratio of successful recognition (RSR) is 94.7% at signal-to-noise ratio (SNR) of −2 dB.

## 1. Introduction

There are considerable interests in radar systems that *"to see and not be seen"* can commonly called low probability of intercept (LPI) radar [1]. LPI radar is used more and more widely. Meanwhile, the number of modulating waveforms is increasing quickly for the LPI radar system. However, it is no longer easy, solely relying on human operators, to recognize the LPI radar of interest. That is, automatic recognition of radar waveform is becoming increasingly important for some critical applications in military-like radar identification, threat analysis, electronic warfare (EW), surveillance, etc. Moreover, it can be applied in civilian areas including signal recognition, spectrum management and cognitive radio, etc.

In the past, many scholars have devoted themselves to exploring the automatic recognition system of the radiation sources in the applications. They have proposed several feasible approaches, making the system more intelligent, more robust, and more like a real human operator. These achievements push forward the development in the field of the signal recognition. In [2] and [3] , the statistical characteristics of the radiation signals is estimated by a maximum likelihood algorithm, and the ratio of successful recognition (RSR) is greater than 90% in the case of signal-to-noise ratio (SNR) ≥ 10 dB. However, the classification of the signals are not extensive, as several kinds of LPI radar waveforms such as Frank code are not mentioned. LPI radar waveform usually has lower signal power, which makes it difficult to classify the LPI radar waveforms directly. Time-frequency (T-F) techniques are used to increase the signal processing gain for LPI radar waveforms [4]. With the application of time-frequency techniques, the T-F feature extraction image processing has become a significant branch of radar waveform recognition. In [5] and [6], Wigner–Ville distribution (WVD) and Pseudo–Wigner distribution (PWD) are adopted to distinguish different frequency modulation (FM) signals, including linear frequency modulation (LFM), FM hyperbolic, FM sinusoidal and others. However, the algorithm for distinguishing polyphase codes (such as Frank code) were not mentioned too much. The short-time Fourier transform (STFT) is presented to analyze the differences between four typical radar signals (LFM, Phase shift keying (PSK), Frequency shift keying (FSK) and continuous wave) in [7] and [8]. In such cases, the RSR is more than 90% at SNR ≥ 0 dB. Nevertheless, it is difficult to distinguish polyphase codes. Zilberman and Pace discussed five kinds of radar waveforms (Binary phase shift keying (BPSK), Frequency modulated continuous wave (FMCW), Frank code, P4 and PT1), who used Choi–Williams time-frequency distribution (CWD) image processing to extract signal features, and the RSR is more than 80% at the SNR of 0 dB [9,10]. Lundén addressed the algorithm which can distinguish eight types of radar waveforms based on Wigner–Ville distribution (WVD) and CWD processing with a high RSR [11]. However, the algorithm estimators of subpulse rate and carrier frequency are needed. In [12], Rihaczek distribution (RD) and Hough transform (HT) are presented to derive two new characteristic features that are especially suitable for the LFM and FSK. However, the RSR of the system reached 90%, in the case that SNR is larger than 24 dB. In [13], three kinds of radar waveforms are recognized (LFM, FSK and PSK), based on random projections and sparse classification, with the RSR over 90% at SNR of 0 dB. Normally, the more kinds of radar waveforms the system identifies, the less robust it is. How to classify more types of radar waveforms in a high noise environment is a challenging problem.

In this paper, an automatic radar waveform recognition system is exploited. Eight types of waveforms (LFM, Costas codes, BPSK, Frank code and P1–P4) were applied in LPI radar, which can identify less but necessary features. Some of the features are obtained from signals directly (we call signal features), and others from a T-F distribution 2D image (we call image features). The signal features consist of second order statistics, power spectral density (PSD) and instantaneous properties (including instantaneous frequency and instantaneous phase), etc. These features are helpful to distinguish polyphase codes from others. Image features are extracted from CWD images of detected signals. CWD is a member of Cohen classes that has fewer cross terms than traditional T-F distributions, such as Wigner–Ville distribution. The extraction procedures from the CWD image includes image binarization, image opening operation, skeleton extraction, Pseudo–Zernike moments [14] and PCA, etc. Image features classify different polyphase codes accurately. After feature extraction, a features selection algorithm based on mutual information, is utilized to pick up more utilitarian features from redundant ones. By applying the greedy selection method, the algorithm selects the features one by one. In other words, the selected features vector is the one that maximizes the mutual information in the classes and the current feature vectors. Elman neural network (ENN) is selected as the classifier cell (*networks*1 and *networks*2) of the recognition system since it is a feedback network which is suitable for the classifier. The whole structure of the classifier that we proposed consists of two cells, and each cell has a three-layer ENN for different classifying purposes. This structure is suitable for the automatic waveform system, which has been seldom mentioned before. It has higher RSR in the low SNR environments and less training time. Experimental results show that the recognition system has total RSR over 94%, when SNR ≥ −2 dB.

Our major contributions are summarized as follows: (1) modifying the type of network of classifier (using Elman neural network instead of traditional neural network), CWD image feature extraction (using skeleton extraction instead of Wigner–Ville distribution, data driven and peak search) provided in Lundén’s approaches and improving the experimental results effectively (see Section 6); (2) creating two original features, ${\hat{\theta }}_{max}$ and ${\hat{a}}_{max}$, which increase the recognition ability; (3) the proposed approaches being classified into eight kinds of waveforms without prior knowledge; and (4) proposing a new classifier structure, with two three-layer Elman neural networks.

The paper is organized as follows. The automatic waveform recognition system and signal model are introduced in Section 2. Section 3 describes the composition of the classifier. Section 4 explains the features that the system required and how to calculate them. After that, a great number of features are calculated. In order to improve the efficiency of the system, the features selection algorithm is given in Section 5. Section 6 shows the simulation results and makes discussions. Finally, Section 7 draws the conclusions.

## 2. System Overview

**Figure 1:**
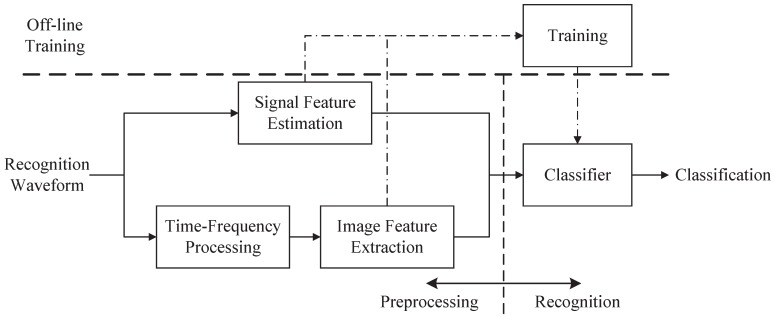
The figure has shown the system components. The adding white noise $\sigma_{\varepsilon }^{2}$ is estimated in signal feature section.

As shown in Figure 1, the system is composed of four modules: time-frequency processing, signal feature estimation, image feature extraction and classifiers. In order to obtain the signal power accurately, it is necessary to estimate the adding of white noise ($\sigma_{\varepsilon }^{2}$) in the samples. Different from majority of the existing systems, the carrier frequency estimation is not required in local systems because the carrier frequency shows the shift of the position in the CWD image. However, the signal structure is not changed at a different frequency, and the advantage of image features is not being sensitive to where the object lies in the image.

**Figure 2:**
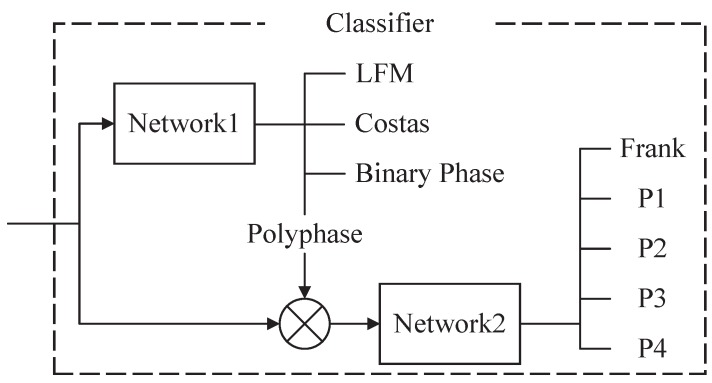
There are two classifier cells, *network*1 and *network*2. *network*2 is controlled by *network*1. When the recognition result is regarded as polyphase by *network*1, *network*2 will start to work. In addition, the input features are classified into five types of polyphase codes directly.

The classifier consists of two classifier cells, *network*1 and *network*2. It can be identified including LFM, Costas codes, BPSK, as well as Frank code and P1–P4 polyphase codes. According to all of signal features and several image features, four types of classifications are classified in *network*1. They are LFM, Costas codes, BPSK and polyphase codes (including Frank and P1–P4). After that, the polyphase codes are classified in *network*2. At the same time, *network*2 is controlled by *network*1. As the signal is considered as polyphase codes by *network*1, *network*2 will begin to work. The reason to design two subsidiary cell structures is to reduce the number of input features and to improve accuracy of the signal classification. For more details, see Figure 2.

In this paper, the center frequency of the signal bandwidth is considered as carrier frequency. In addition, the signal is disturbed by additive white Gaussian noise, which means that the intercepted discrete time signal model is given by

(1) $$y\left(nT\right)=s\left(nT\right)+m\left(nT\right)=Ae^{j\phi (nT)}+m\left(nT\right)$$

where *n* is an integer, *T* is a sampling interval, s(nT) is the complex of the transmitted signals, and m(nT) is complex white Gaussian noise with the variance $\sigma_{\varepsilon }^{2}$. *A* is amplitude, for simplicity and without loss of generality, so we assume that *A* is an invariant constant. ϕ is the instantaneous phase of the complex signal. If the input signal is real, the Hilbert transform is used:

(2) y(k)=x(k)+jH[x(k)]

where x(k) is the real signal, and H[·] is Hilbert transform. For more details, see [15].

## 3. Waveform Classifier

**Figure 3:**
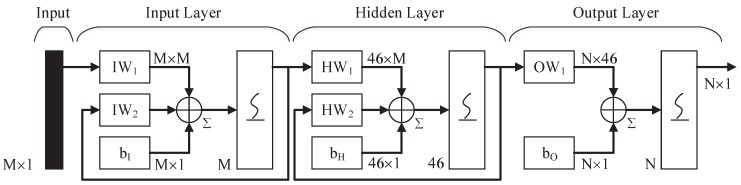
There are three layers in the Elman neural network. The neurons of input layers are determined by the number of input features. The output layers are decided by the classification. The hidden layer contains 46 neurons as well.

The three-layer ENN is used for the signal classification in the paper. There are connections from the hidden layer to these context units fixed with a weight of one [16]. At each time step, the input is propagated in a standard feed-forward fashion, and then the error back propagation (BP) learning rule is applied [17]. The fixed back connections result in context units always maintaining a copy of previous values of hidden units (since they propagate over the connections before the BP learning rule is applied). Thus, the network can maintain a sort of state, allowing it to perform such tasks as sequence-prediction that are beyond the power of a standard multi-layer perceptron [18,19]. In Figure 3, the hidden layer contains 46 neurons, sigmoid activation $f\left(x\right)=1/\left(1+e^{-x}\right)$ is selected in each layer, and the number of input and output layers’ neurons are determined by the vector dimension, respectively.

For ENN, a method to fix the number of hidden layer neurons $H_{num}$ is discussed in [20]. A simple calculation formula is formulated as:

(3) $$H_{num}=\frac{C\times X+0.5\times C\times (X^{2}+X)-1}{C+X}$$

where *C* is the number of classification, and *X* is the dimension of a feature vector obtained. A slight adjustment of the number of hidden layer neurons needs to be done, until the network achieves the optimal situation. A simple suggestion is to re-enter the training data as the test data, and the correct recognition rate should be greater than 99%. Otherwise, the number of hidden layer neurons should be adjusted. Forty-six neurons are used in this paper.

## 4. Features Extraction

In this section, the feature extraction is described in details. Feature extraction plays a key role in the automatic recognition system, the algorithm of which determines not only the RSR but also the robustness of the system. The features include signal features and image features. The section is organized as follows. First, the signal features that based on the second order statistics, PSD, and instantaneous properties are illuminated. Then, the Choi–Williams distribution is introduced, and a further eight types of radar waveforms are shown in CWD images, respectively. After that, image preprocessing based on image morphology is addressed. Finally, the image features of the waveforms are estimated and extracted from the CWD image.

Table 1 lists the necessary features that are presented in *network*1 and *network*2. Meanwhile, in order to keep the classifiers as concise as possible, other features are considered but not listed. For example, higher order moments (up to the fourth order) and cumulants (up to the fourth order), Pseudo–Zernike moments (up to the eighth order) and other instantaneous properties, etc. However, these features are not found discriminative enough in the system recognition. How to select the final features will be described in Section 5.

### 4.1. Signal Features

#### 4.1.1. Based on Second Order Statistics

The *n*th order moment of the complex signal is given by

(4) $${\hat{M}}_{nm}=\left|\frac{1}{N}\sum_{k=0}^{N-1}y^{n-m}\left(k\right){\left(y^{*}\left(k\right)\right)}^{m}\right|$$

where *N* is the number of samples, and (∗) is conjugated components. ${\hat{M}}_{10}$ and ${\hat{M}}_{20}$ are calculated from this formula. The absolute value is ensured that the estimate value is an invariant constant when the signal phase rotated.

The *n*th order cumulant is given by [21,22]

(5) $${\hat{C}}_{nm}=\left|\frac{1}{N}\sum_{k=0}^{N-1}{\left(y\left(k\right)-{\hat{M}}_{10}\right)}^{n-m}{\left(y^{*}\left(k\right)-{\hat{M}}_{10}\right)}^{m}\right|$$

where ${\hat{M}}_{10}$ is given in Equation (4). In the system, ${\hat{C}}_{20}$ is calculated. Additive independent complex second order circular noise does not affect moment features and cumulant features.

#### 4.1.2. Based on Power Spectral Density (PSD)

PSD describes how the signal power distributes in frequency domain. Two features based on PSD are utilized. The signal should be estimated as an invariant scaling before PSD is calculated. Therefore, invariant scaling is estimated as [23]:

(6) $$\tilde{y}\left(k\right)=\frac{y(k)}{\sqrt{{\hat{M}}_{21}-\sigma_{\varepsilon }^{2}}}$$

where y(k) is the *k*th of samples, and ${\hat{M}}_{21}$ is the three-order moment obtained in Equation (4). The variance of the additive noise $\sigma_{\varepsilon }^{2}$ can be obtained in [24].

The features about PSD are given by

(7) $$\gamma_{m}=\frac{1}{N}\max_{n}\;\left\{\frac{1}{N}{\left|\sum_{k=0}^{N-1}{\tilde{y}}^{m}\left(k\right)e^{-j2\pi nk/N}\right|}^{2}\right\}$$

where $\tilde{y}\left(k\right)$ is given in Equation (6). $\gamma_{1}$ is a nice feature to distinguish between binary phase and Costas codes. In fact, the square of the complex envelope is constant for binary phase modulation signals. The feature $\gamma_{2}$ makes an outstanding performance in distinguishing binary phase modulation signals from others.

#### 4.1.3. Based on Instantaneous Properties

Instantaneous properties, which include enormous information, work well in distinguishing frequency modulation signal from phase modulation signal. In this paper, instantaneous frequency and instantaneous phase need to be estimated. The standard deviation of the absolute value of instantaneous phase is estimated as [25]. For the sake of simplicity, $\phi \left(k\right)={\text{tan}}^{-1}\left[Im\left(y\left(k\right)\right)/Re\left(y\left(k\right)\right)\right]$ is utilized, where Im and Re are the imaginary and real parts of the signal, respectively:

(8) $${\hat{\sigma }}_{\phi }=\sqrt{\frac{1}{N}\left(\sum_{k=0}^{N-1}\phi^{2}\left(k\right)\right)-{\left(\frac{1}{N}\sum_{k=0}^{N-1}\left|\phi (k)\right|\right)}^{2}}$$

where *N* is the number of samples, ϕ is the instantaneous phase of the complex signal, and the range of ϕ is between −*π* and *π*. For more details, see [3].

In order to estimate the instantaneous frequency clearly, the method is decomposed into several steps:

- calculate ϕ(k);
- compute ${\phi_{u}\left(k\right)}^{*}$ from ϕ(k);
- set ${f(k)}^{**}=\phi_{u}\left(k\right)-\phi_{u}\left(k-1\right)$;
- calculate $\mu_{f}:=\frac{1}{N}\sum_{k=0}^{N-1}f(k)$;
- calculate the normalized centered instantaneous frequency $\tilde{f}\left(k\right)$, i.e.;
  $$\tilde{f}\left(k\right)=\left(f\left(k\right)-\mu_{f}\right)/\left(\max\left|f\left(k\right)-\mu_{f}\right|\right)$$
- the absolute value of the normalized centered instantaneous frequency is given by;
  (9) $${\hat{\sigma }}_{f}=\sqrt{\frac{1}{N}\left(\sum_{k=0}^{N-1}{\tilde{f}}^{2}\left(k\right)\right)-{\left(\frac{1}{N}\sum_{k=0}^{N-1}\left|\tilde{f}\left(k\right)\right|\right)}^{2}}$$

* $\phi_{u}\left(k\right)$ corrects the radian phase angles in ϕ(k) by adding multiples of ±2π when absolute jumps between consecutive element of ϕ(k) is greater than or equal to the jump tolerance of *π* radians.
** There are some spikes in the instantaneous frequency estimation in the vicinity of phase discontinuity of some waveforms. In order to smooth the f(k) and to remove the spikes, a median-filter with window size 5 is used.

### 4.2. Choi–Williams Distribution (CWD)

Choi–Williams distribution is a member in Cohen Classes [26], which can reduce image interference from cross terms effectively:

(10) $$\begin{array}{cc} C(t,\omega )= & {\int \int \int }_{\infty }e^{j2\pi \xi (s-t)}f\left(\xi ,\tau \right)\\ & \cdot x\left(s+\tau /2\right)x^{*}\left(s-\tau /2\right)e^{-j\omega \tau }d\xi dsd\tau \end{array}$$

where *t* and *ω* are time and frequency axes, and f(ξ,τ) is the kernel function given by

(11) $$f\left(\xi ,\tau \right)=\exp\left[\frac{{\left(\pi \xi \tau \right)}^{2}}{2\sigma }\right]$$

**Figure 4:**
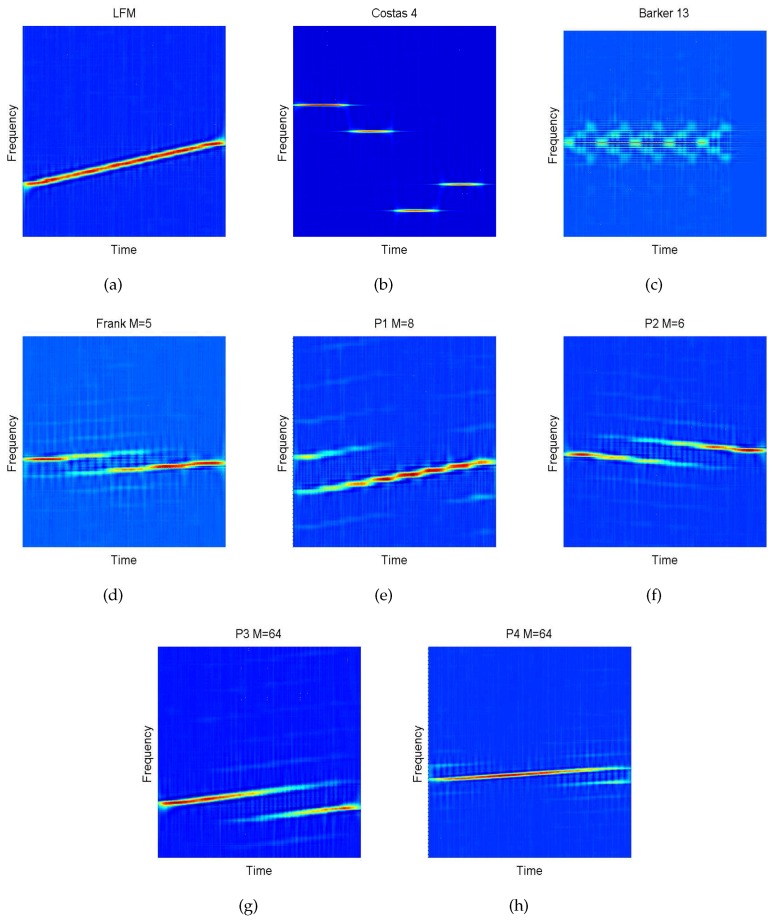
In this figure, (**a**)–(**h**) are different waveform classes, which including Linear frequency modulation (LFM), Costas codes, Binary phase shift keying (BPSK), Frank, P1, P2, P3 and P4 sequentially. There are significant differences among the Choi–Williams time-frequency distribution (CWD) images. The controllable factor σ=1 also be used.

The kernel function is a low-pass filter to eliminate cross terms. *σ* refers to controllable factor. The bigger *σ* is, the more obvious cross terms are. Meanwhile, σ=1 is used in this paper to balance the cross terms and resolution. Eight types of waveforms of CWD transformation are shown in Figure 4. A method for fast calculation of CWD is found in [10]. The structure of a fast CWD method is based on the standard fast Fourier transformation (FFT). Therefore, the number of sampling points is recommended to be a small power of two, such as 128, 256, 512, etc. In this paper, 1024 × 1024 points are selected.

### 4.3. Image Preprocessing

In the following parts, the real parts of CWD results of waveforms is treated as a 2D image. Digital image processing is explored to gain interested features. In this part, CWD image is processed into a binary image with three operations.

First, the length of detected signal, however, is N<1024 points in most cases. Zero padding is utilized because we select the CWD transformation of 1024 points. Then, the CWD image is resized to N×N to reduce the computation load. Finally, the resized image is converted to a binary image, based on global thresholding algorithm [27]. The operation steps are as following:

- transform the resized image to gray image between [0,1], i.e.;
  $$G\left(x,y\right)=\frac{{CWD}_{N\times N}\left(x,y\right)-\min{CWD}_{N\times N}\left(x,y\right)}{\max({CWD}_{N\times N}\left(x,y\right)-\min{CWD}_{N\times N}\left(x,y\right))}$$
- estimate the initial threshold *T*. It can be obtained from the average of the minimum and maximum from the image G(x,y);
- divide the image into two pixel groups $G_{1}$ and $G_{2}$ after the comparison with the threshold *T*. $G_{1}$ includes all pixels in the image that the values >T, and $G_{2}$ includes all pixels in the image that the values ≤T ;
- calculate the average value $\mu_{1}$ and $\mu_{2}$ of two pixel groups $G_{1}$ and $G_{2}$, respectively;
- update the threshold value;
  $$T=\frac{\mu_{1}+\mu_{2}}{2}$$
- repeat (b–e), and calculate δT, i.e.;
  $$\delta T=T_{now}-T_{before}$$
- until the δT is smaller than a predefined convergence value, 0.001 is used in the paper;
- calculate B(x,y);
  $$B\left(x,y\right)=\left\{\begin{array}{ll} 1 & G(x,y)\geq T\\ 0 & others \end{array}\right.$$
- output the final binary image B(x,y).

**Figure 5:**
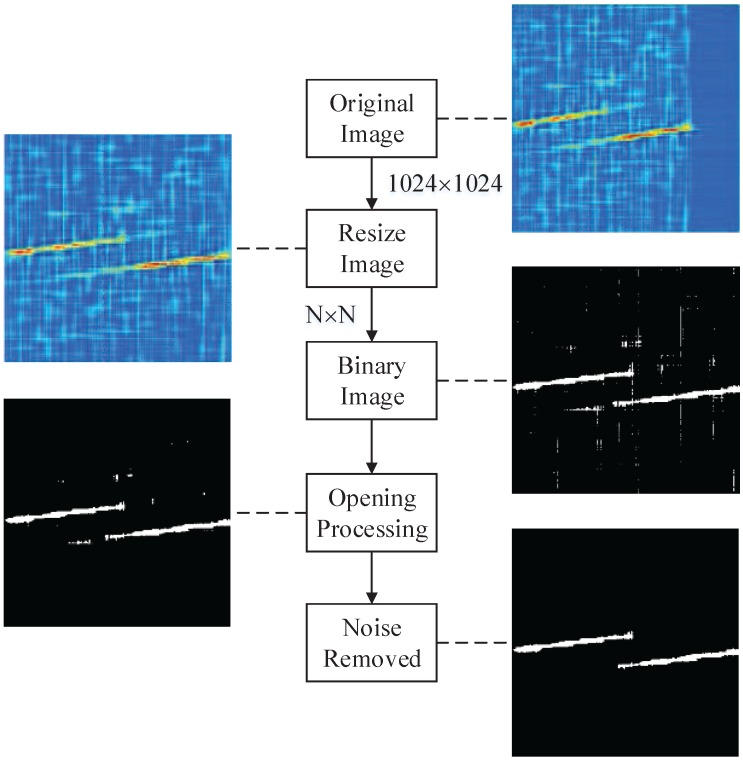
In this figure, P3 code is selected at signal-to-noise ratio (SNR) of –6 dB as an example. Original image is resized by N×N, and the bicubic interpolation is adopted. That is, the output pixel value is a weighted average of pixels in the nearest 4-by-4 neighborhood. By using the opening processing, the binary image is smoother and the noise is removed in the last.

After the image binarization, however, there is some isolated noise and processing noise in the binary images. Isolated noise is generated because the signal is transmitted in the noisy environment. In addition, processing noise is generated in the kernel of CWD itself. It is a kind of special straight line, thin but long. The width of the line is less than three pixels, whereas the majority of lines in the CWD image are longer than half of the image length. In order to remove the noise, a morphological opening is applied (erosion followed by dilation). Erosion and dilation are the basic operations in morphological image processing. Morphological techniques probe an image with a small shape or template called a structuring element. The structuring element is positioned at all possible locations in the image. Furthermore, it is compared with the corresponding neighbourhood of pixels. The structuring element is said to fit the image if, for each of its pixels set to 1, the corresponding image pixel is also 1. Similarly, a structuring element is said to hit, or intersect, an image if, at least for one of its pixels set to 1, the corresponding image pixel is also 1. The opening is so called because it can open up a gap between objects connected by a thin bridge of pixels. Any regions that have survived from the erosion are restored to their original size through the dilation. In the paper, the structuring element in the size of 3 × 3 pixels is used. After the opening operation, the groups as small as a minimum 10% of the size of the largest group are removed. The image process is introduced in Figure 5.

### 4.4. Image Features

The number of objects in the binary image ($N_{obj}$) is a useful feature. For example, Frank code and P3 have two objects, respectively, while LFM and P1 only have one. However, Costas codes have many objects in different location. In order to distinguish different waveforms, two features $N_{obj1}$ and $N_{obj2}$ are used in the paper. $N_{obj1}$ and $N_{obj2}$ are the number of the objects that represent larger than 20% and 50% of the size of the largest object, respectively.

A feature is also found in the location of the maximum energy in time domain of the image, i.e.,

(12) $$t_{max}=\frac{1}{N-1}\arg\max_{t}\;\left\{C_{N\times N}\left(t,\omega \right)\right\}$$

where $C_{N\times N}\left(t,\omega \right)$ is a resized version of the CWD image, and *N* is the length of the sampling data.

The standard deviation of the width of the signal objects (${\hat{\sigma }}_{obj}$) and the rotate angle of the largest object (${\hat{\theta }}_{max}$) are appropriate for polyphase codes discrimination. Namely, the feature ${\hat{\sigma }}_{obj}$ is suitable to classify two kinds of waveforms such as “stepped waveform” (including Frank code, P1) and “linear waveform” (including LFM, P3 and P4). In eight types of waveforms, only P2 has a negative slope. Therefore, P2 can be picked out by the parameter ${\hat{\theta }}_{max}$ from others easily. The features are calculated as follows:

- for each object, $k=1,2,\ldots ,N_{obj1}$;
  - decide the *k* and mask the other objects away from the binary image;
  - calculate the principal components of the binary image;
  - rotate^∗^ the image until the principal component of the object is parallel to the vertical axis, recorded as $B_{r}\left(x,y\right)$;
  - calculate the row sum, i.e., $r\left(x\right)=\sum_{y=0}^{N-1}B_{r}\left(x,y\right)$, x=0,1,2,…,N−1;
  - normalize r(x), i.e., $\hat{r}\left(x\right)=r\left(x\right)/\max\left\{r\left(x\right)\right\}$;
  - calculate the standard deviation of $\hat{r}\left(x\right)$, i.e.,
    $${\hat{\sigma }}_{k,obj1}=\sqrt{\left(1/N\right)\sum_{x}{\hat{r}}^{2}\left(x\right)-{\left(\left(1/N\right)\sum_{x}\hat{r}\left(x\right)\right)}^{2}}$$
    where *N* is the number of samples.
- output the rotation degree of the maximum of objects ${\hat{\theta }}_{max}$;
- output the average of the ${\hat{\sigma }}_{k,obj1}$, i.e.;
  (13) $${\hat{\sigma }}_{obj}=\left(\sum_{k=1}^{N_{obj1}}{\hat{\sigma }}_{k,obj1}\right)/N_{obj1}$$

* nearest neighbor interpolation is used in rotation processing.

Next, we select the maximum object with others removed, extracting the skeleton of the maximum object and estimating the linear trend of it. In the estimation, minimizing the square errors method is applied. The linear trend is subtracted from skeleton of the object to acquire the resulting vector $f_{n}$. The standard deviation of $f_{n}$ is given by

(14) $${\hat{\sigma }}_{Wf}=\sqrt{\frac{1}{M-1}\sum_{k=1}^{M}f_{n}^{2}\left(k\right)-{\left(\frac{1}{M-1}\sum_{k=1}^{M}f_{n}\left(k\right)\right)}^{2}}$$

where *M* is the length of $f_{n}$.

In order to express the randomness of $f_{n}$, a statistical characteristics test is proposed. The details of the test are as follows:

- calculate the average of $f_{n}$;
- calculate b(k) as follows $b\left(k\right)=\left\{\begin{array}{cc} 1 & f_{n}\left(k\right)>{\bar{f}}_{n}\\ 0 & others \end{array}\right.$, k=1,…,N;
- a consecutive sequence of 0’s or 1’s is called a unit, and *R* is the number of units. Let $N_{A}$ and $N_{B}$ denote the statistics number of b(k)=1 and b(k)=0, respectively, i.e., $N_{A}+N_{B}=N$;
- calculate the mean of units, i.e.;
  $$E\left(R\right)=\left(N+2N_{A}N_{B}\right)/N$$
- calculate the variance of units, i.e.;
  $$Var\left(R\right)=\frac{2N_{A}N_{B}\left(2N_{A}N_{B}-N\right)}{N^{2}\left(N-1\right)}$$
- calculate the value of test statistic *Y*, i.e.;
  Y=(R−E(R))/Var(R)
- output the probability feature, i.e.;
  (15) $${p_{R}}^{*,**}=2(1-\Phi (|Y|))$$

* where Φ(·) is the standard normal cumulative distribution function. The value of $p_{R}$ is between 0 and 1.
** note that $p_{R}$ is no longer a probability. It is a measure of the similarity with Gaussian distribution. The standard deviation $\sqrt{Var(\cdot )}$ value is too small for machine precision. Therefore, it is replaced by variance Var(·) in (f).

When the $p_{R}$ is closer to 1, the test signal is more similar to Gaussian distribution, and the number of *N* is more than 50, the distribution of *R* is similar to the standard normal distribution, i.e., $Z=\left(R-E\left(R\right)\right)/\sqrt{Var(R)}∼N\left(0,1\right)$. However, the values of Var(R) are quite small for machine precision for the P4 codes. Therefore, the value of *R* is normalized by using the equation Y=(R−E(R))/Var(R). The feature $p_{R}$ is worked in *network*2 only.

**Figure 6:**
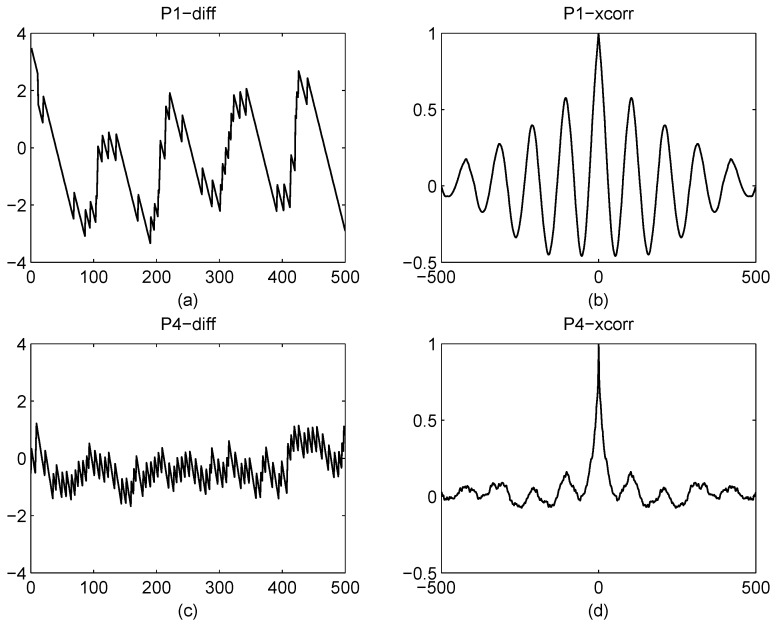
In this figure, (**a**) and (**c**) are resulting vector $f_{n}$ of P1 and P4, respectively. (**b**) and (**d**) are the autocorrelation of P1 and P4. The autocorrelation of P1 has a strong oscillation, similar to a periodic signal. However, the autocorrelation of P4 has a random oscillation, similar to a white noise signal.

The other three features are obtained from the autocorrelation of $f_{n}$, i.e., $c\left(m\right)=\sum_{k}f_{n}\left(k\right)f_{n}\left(k-m\right)$, and m=0,1,…,N−1. Figure 6 indicates the differences of P1 and P4. In order to characterize these differences, the features are introduced.

The ratio of maximum and sidelobe maximum, i.e.,

(16) $$r=\frac{N\max_{m\in [m_{0},N-1]}c\left(m\right)}{\left(N-m_{1}\right)\max c\left(m\right)}$$

where, $m_{0}$ is the location of the minimum of the lag value, and $m_{1}$ is the location of the sidelobe maximum of the lag value in the $\left[m_{0},N-1\right]$. The FFT result is selected to characterize the power of oscillation of c(m), and the feature is estimated as:

(17) $${\hat{a}}_{max}=\max\left\{\text{abs}\left(\text{FFT}\left(c\left(m\right)\right)\right)\right\}$$

where c(m) is normalized by using the maximum value of itself. Namely, the range of c(m) is [−1,1].

The final features are the members of Pseudo–Zernike moments. Pseudo–Zernike moments are invariant to translation, rotation, scaling and mirroring. It is suitable for the problems about pattern recognition [28,29,30]. These invariant features can reduce the amount of data used in training, which makes the recognition easy. The p+q order of the image geometric moments are defined as:

(18) $$m_{pq}=\sum_{x}\sum_{y}B\left(x,y\right)x^{p}y^{q}$$

where B(x,y) is the binary image. The scaling and translation invariant central geometric moments are given by

(19) $$G_{pq}=\frac{1}{m_{00}^{(p+q+2)/2}}\sum_{x}\sum_{y}B\left(x,y\right){\left(x-\bar{x}\right)}^{p}{\left(y-\bar{y}\right)}^{q}$$

where $\bar{x}=m_{10}/m_{00}$ and $\bar{y}=m_{01}/m_{00}$.

The scaling and translation invariant radial geometric moments are given by

(20) $$R_{pq}=\frac{1}{m_{00}^{(p+q+3)/2}}\sum_{x}\sum_{y}B\left(x,y\right){\left({\tilde{x}}^{2}+{\tilde{y}}^{2}\right)}^{1/2}{\tilde{x}}^{p}{\tilde{y}}^{q}$$

where $\tilde{x}=x-\bar{x}$ and $\tilde{y}=y-\bar{y}$.

The Pseudo–Zernike moments are defined as:

(21) $$\begin{array}{cc} Z_{nm}= & \frac{n+1}{\pi }\sum_{\frac{s=0}{n-s-m=even}}^{n-|m|}\sum_{a=0}^{k}\sum_{b=0}^{m}{\left(-j\right)}^{b}\\ & \left(\begin{array}{c} k\\ a \end{array}\right)\left(\begin{array}{c} m\\ b \end{array}\right)D_{nms}G_{2k-2a+m-b,2a+b}\\ & +\frac{n+1}{\pi }\sum_{\frac{s=0}{n-s-m=odd}}^{n-|m|}\sum_{a=0}^{d}\sum_{b=0}^{m}{\left(-j\right)}^{b}\\ & \left(\begin{array}{c} d\\ a \end{array}\right)\left(\begin{array}{c} m\\ b \end{array}\right)D_{nms}R_{2d-2a+m-b,2a+b} \end{array}$$

where k=(n−s−m)/2, d=(n−s−m−1)/2 and

(22) $$D_{nms}={\left(-1\right)}^{s}\frac{(2n+1-s)!}{s!(n-|m|-s)!(n+|m|+1-s)!}$$

The dynamic range can be reduced by calculating the logarithm, i.e., ${\hat{Z}}_{nm}=\text{ln}\left|Z_{nm}\right|$. The members of the Pseudo–Zernike moments, including ${\hat{Z}}_{20}$, ${\hat{Z}}_{22}$, ${\hat{Z}}_{30}$, ${\hat{Z}}_{31}$, ${\hat{Z}}_{32}$, ${\hat{Z}}_{33}$ and ${\hat{Z}}_{43},$ are selected. The features are used in *network*2 only because the features in the *network*1 can distinguish between LFM, Costas codes, BPSK and polyphase codes very well.

## 5. Features Selection

In this section, the features selection algorithm for final feature vectors is exploited. With the increase of dimension of feature vector, the redundant information should be removed. The algorithm can select excellent features from a large number of them. Thus, the training time is reduced [31]. Based on the mutual information in the features, greedy selection algorithm is selected. It means that a new feature that has the maximum of mutual information is joined to the feature vector (which includes the previously selected features) in each cycle. The procedure is continued until the number of selected features reaches our design. Mutual information is estimated between class *C* and the feature vectors ***X***:

(23) $$\begin{array}{cc} \hat{I}\left(X;C\right) & =H\left(C\right)-\hat{H}\left(C|X\right)\\ & =-\sum_{k=1}^{c}p\left(c_{k}\right)\log p\left(c_{k}\right)\\ & \;+\frac{1}{N}\sum_{j=1}^{N}\sum_{k=1}^{c}\hat{p}\left(c_{k}|x_{j}\right)\log\hat{p}\left(c_{k}|x_{j}\right) \end{array}$$

where $c_{k}$ is the *k*th class and *c* is the number of classes. H(·) denotes the entropy, *N* is the total number of training data and $x_{j}$ is the *j*th training vector. Probability density function $\hat{p}\left(c_{k}|x_{j}\right)$ is estimated by using Parzen windows. Entropy of the class H(C) is calculated by using the training data, i.e., $p\left(c_{k}\right)=N_{k}/N$, where $N_{k}$ is the total number of training data from class $c_{k}$. The $\hat{p}\left(c_{k}|x_{j}\right)$ is given by

(24) $$\hat{p}\left(c_{k}|x_{j}\right)=\frac{\sum_{i\in c_{k}}\exp(-\frac{{\left(x_{j}-x_{i}\right)}^{T}\Delta^{-1}\left(x_{j}-x_{i}\right)}{2h^{2}})}{\sum_{k=1}^{c}\sum_{i\in c_{k}}\exp(-\frac{{\left(x_{j}-x_{i}\right)}^{T}\Delta^{-1}\left(x_{j}-x_{i}\right)}{2h^{2}})}$$

where Δ is a covariance matrix of a *d*-dimensional vector, and *h* is the window width parameter, (i.e., $h=1/\log_{2}\left(n\right)$), in which, *n* is the number of samples. For more details, see [31].

**Figure 7:**
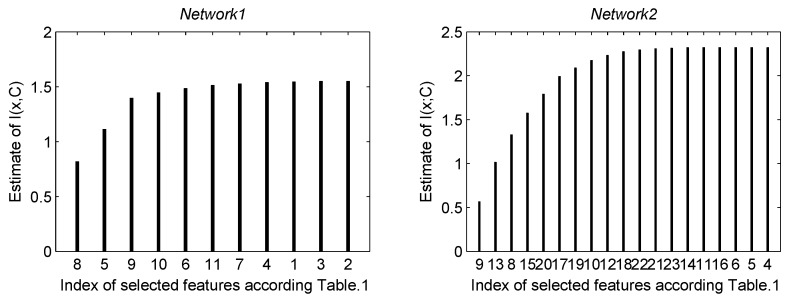
In this figure, the features were selected by the mutual information. The abscissa was the index of selected features at each cycle. There was one-to-one correspondence between the index and the Table 1. For example, the 5th feature was selected at the second cycle in *network*1. It indicated that the 5th feature ($\gamma_{2}$) had the greatest mutual information in all of the features except the 8th feature ($N_{obj1}$). Its ordinate was the sum mutual information of $N_{obj1}$ and $\gamma_{2}$. In *network*1, 11 features were used, and the H(C)=1.5488. In addition, in *network*2, 19 features were used, and H(C)=2.3219.

In this stage, a total of 23 features for *network*1 and *network*2 are selected. The mutual information between each individual feature and class are estimated. In addition, there are 10 and 19 features remaining for *network*1 and *network*2, respectively. The mutual information estimation at each step of the features selection algorithm for *network*1 and *network*2 are shown in Figure 7. However, features selection would be stopped, when the mutual information less than 10^−4^ because the estimation of mutual information is not very accurate especially in a high dimension [32]. See Figure 7 and Table 1 for the list of selected features.

## 6. Simulation and Discussion

In this section, the automatic waveform recognition system is measured by simulation signals. The purpose is to test accurate rate of the identification results, robustness and computational complexity in different conditions.

### 6.1. Create Simulation Signals

In this part, we create the simulation signals for experiments. In order to expedite the system recognition, the continuous sampling points are intercepted from the observation window. Supposing radar sources are disturbed by additive white Gaussian noise (AWGN) in propagation channel. In addition, SNR is defined as $\text{SNR}=10\log_{10}\left(\sigma_{s}^{2}\right)/\left(\sigma_{\varepsilon }^{2}\right)$, where $\sigma_{s}^{2}$ and $\sigma_{\varepsilon }^{2}$ are variances of original signal and noise respectively. U(·) denotes a uniform frequency. For example, there is a original frequency $f_{0}=1000$, and the sample rate $f_{s}=8000$. The uniform result is $f_{0}=U\left(f_{0}/f_{s}\right)=U\left(1/8\right)$. For every waveform, there are different parameters that need to be set. For LFM, the initial frequency is a random number between U(1/16) and U(1/8). Bandwidth Δf ranges from U(1/16) to U(1/8) as well. For BPSK, the random value of the carrier frequency is U(1/8,1/4), and the length of Barker codes selects from {7,11,13}. The number of code periods $\left(N_{p}\right)$ and the number of cycles per phase code (cpp) are range of [100,300] and [1,5], respectively. For Costas codes, the number of frequency change is a random value between 3 and 6. The fundamental frequency $f_{min}$ is U(1/24). For instance, once the Costas codes are selected, the number is 4, namely, a no-repeating sequence {3,2,1,4} is generated. Then, the Costas codes $\left\{3f_{min},2f_{min},f_{min},4f_{min}\right\}$ is decided. For Frank code, the carrier frequency is U(1/8,1/4). In addition, cpp and *M* are range of [1,5] and [4,8], respectively. For P1–P4 polyphase codes, the parameters are similar to Frank code. For more details, see Table 2.

### 6.2. Experiment With SNR

**Figure 8:**
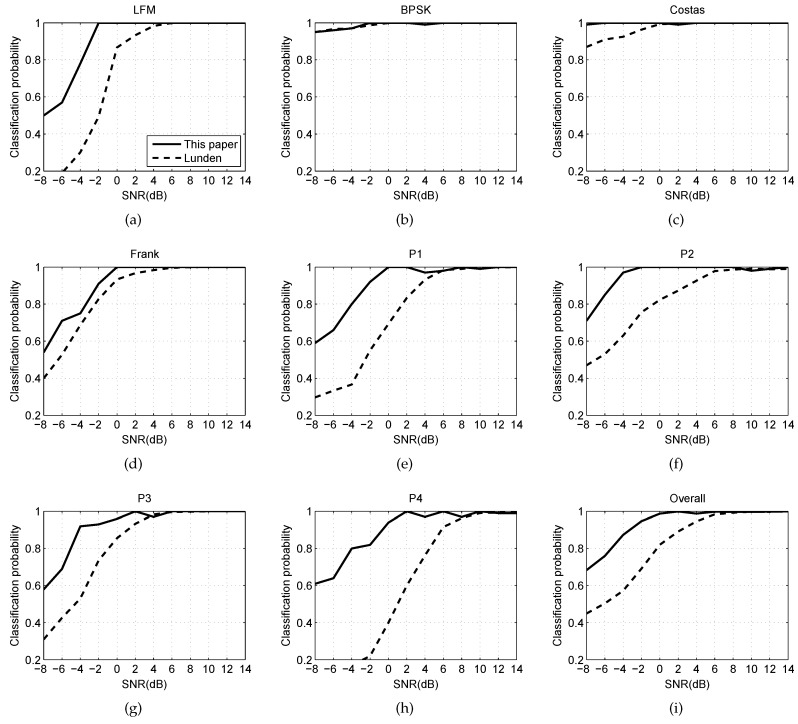
In this figure, (**a**)–(**h**) are the probabilities of 8 types of radar waveforms that measured by the testing data respectivel; (**i**) is the successful rate of overall waveforms.

The experiment research shows the relationship between the RSR and SNR. One thousand groups are provided for every type of radar waveform, from which 90% of the groups data are used for training and 10% for testing. The SNR would be increased from −8 dB to 14 dB with a step of 2 dB. The system will compare with Lundén’s [11] because his system has similar classifications to us, and also is considered as the satisfactory automatic radar classification system at present. The simulation results are shown in Figure 8.

Figure 8 plots the classification probabilities as a function of the SNR. The probabilities are measured by the testing data of each kind of waveforms, and the overall probabilities are also calculated. As shown in Figure 8, our system performs better on the classification of each kind of waveform than Lundén’s, especially at low SNR. At SNR > 8 dB, RSR of our system approaches 100%. With the decrease of SNR, the successful ratio can still be kept at a high level. However, the performance of Lundén’s is far from satisfactory. At SNR of −2 dB, most waveforms of RSR reach 90%. Moreover, the overall RSR of the waveforms approach 95%. Nevertheless, the overall RSR of Lundén’s is less than 70% in the same conditions. Costas codes and BPSK are not sensitive with SNR, and P4 code has low RSR in the simulations. Table 3 shows the confusion table at SNR of −2 dB, while the overall RSR is 94.7%. As the table shows, the probabilities of error recognition of Frank code and P4 code are higher than others. Some of Frank codes are identified as P3 codes by the system, while P4 codes are identified as LFM. In fact, the two pairs are very similar, which can be seen in Figure 4. When the SNR is low, the probabilities of error recognition is also reasonable.

### 6.3. Experiment with Robustness

In this part, we explore the robustness of approach through a different number of training samples. There are 200 groups of testing samples for each of the radar waveform, and the number of training samples would be increased from 100 to 900 with a step of 200. To be more adequate, the experiment would be repeated three times with samples from −8 dB, −2 dB and 10 dB.

**Figure 9:**
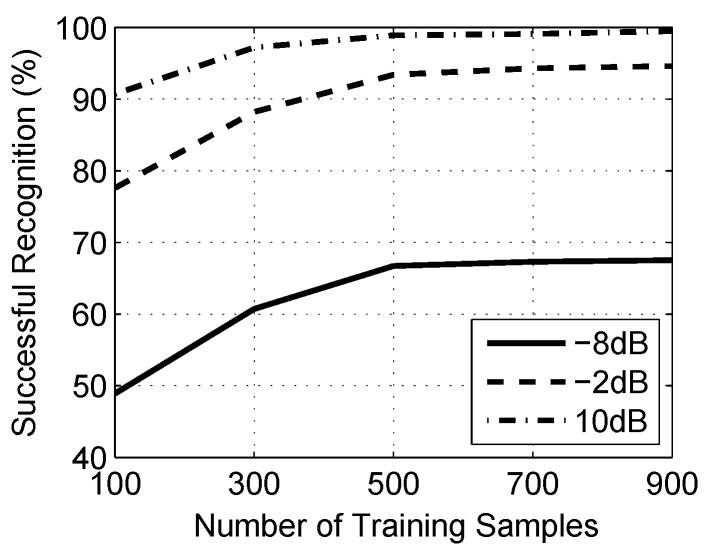
In this figure, successful recognition is measured by a different number of training samples.

As shown in Figure 9, with the increase in number of training samples, the successful recognition is also increased. Meanwhile, for all groups of SNR, the change of successful recognition is not obvious with the number over 500. It means that as long as the number is over 500, the classifier will be able to work in the best state. The approach works well in the small number of samples, which has important significance for radar waveform analysis.

### 6.4. Experiment with Computation

Computational complexity issue is an important indicator to measure the the performance of classification system. We reproduce Lundén’s method [11], and compare it with this paper in the same conditions. All eight kinds of waveforms are tested under three different SNRs: −8 dB, −2 dB and 10 dB, and each test repeats 10 times on average. The testing environment and testing results are demonstrated as Table 4 and Table 5, respectively.

As shown in Table 5, the proposed method spends about 55 s and Lundén’s about 85 s, respectively. However, Lundén’s method has several approaches which do not apply in this paper, such as Wigner–Ville distribution, data driven and peak search, etc. These approaches consume extra time. In each waveform, there is the trace of reduction in time, when SNR is increasing. BPSK spends the least time and P3 spends the most time, but in the overall range, the difference is not obvious. The recipe in this paper is stable, and the variety of SNR and waveforms have little effect on the computational complexity of system.

## 7. Conclusions

In this paper, an automatic radar waveform recognition system is explored. The recognition system can identify eight types of LPI radar waveforms (including LFM, Costas codes, BPSK (Barker modulation), Frank code and P1–P4) in a highly noisy environment. The RSR relies more on the choice of the features extracted from an intercepted radar pulse as well. The features include two categories, one is extracted from waveforms directly (signal features), the other is from time-frequency image (image features). To reduce cross terms, the CWD transformation is adopted in the time-frequency image. The final feature vectors have been selected by using the information theoretic features selection algorithm.

Elman neural network, a typical global network, is applied as the classifier cell of the recognition system. It is a robust network that has a memory function. The structure of the classifier is composed of two cells, *network*1 and *network*2. In *network*1, the waveforms are classified into four types (including LFM, Costas codes, BPSK and polyphase codes), through the use of all signal features and a part of image features. The polyphase codes are further classified into five types (including Frank code, P1–P4) in *network*2.

New features have been proposed from the time-frequency image. By adopting translation, scaling and rotation features, we can focus more on the structure of the waveform itself. It is important to classify different kinds of polyphase codes and improve the RSR of polyphase codes. The simulation results show when SNR ≥ −2 dB, and the RSR is more than 94%.

## Figures and Tables

**Table 1:** List of features selected for the networks.

Index	Features	*Network1*	*Network2*
1	Moment ${\hat{M}}_{10}$	✔	
2	Moment ${\hat{M}}_{20}$	✔	
3	Cumulant ${\hat{C}}_{20}$	✔	
4	PSD maximum $\gamma_{1}$	✔	✔
5	PSD maximum $\gamma_{2}$	✔	✔
6	Std. of instantaneous phase ${\hat{\sigma }}_{\phi }$	✔	✔
7	Std. of instantaneous freq. ${\hat{\sigma }}_{f}$	✔	
8	Num. of objects (10%) $N_{obj1}$	✔	✔
9	Num. of objects (50%) $N_{obj2}$	✔	✔
10	CWD time peak location $t_{max}$	✔	✔
11	Std. of object width ${\hat{\sigma }}_{obj}$	✔	✔
12	Maximum of PCA degree ${\hat{\theta }}_{max}$		✔
13	Std. of $f_{n}$ ${\hat{\sigma }}_{Wf}$		✔
14	Statistics test $p_{R}$		✔
15	Autocorr. of $f_{n}$ *r*		✔
16	FFT of corr. $f_{n}$ ${\hat{a}}_{max}$		✔
17	Pseudo–Zernike moment ${\hat{Z}}_{20}$		✔
18	Pseudo–Zernike moment ${\hat{Z}}_{22}$		✔
19	Pseudo–Zernike moment ${\hat{Z}}_{30}$		✔
20	Pseudo–Zernike moment ${\hat{Z}}_{31}$		✔
21	Pseudo–Zernike moment ${\hat{Z}}_{32}$		✔
22	Pseudo–Zernike moment ${\hat{Z}}_{33}$		✔
23	Pseudo–Zernike moment ${\hat{Z}}_{43}$		✔

**Table 2:** List of simulation parameters.

Radar Waveforms	Parameter	Ranges
-	Sampling rate $f_{s}$	1
LFM	Initial frequency $f_{0}$	U(1/16,1/8)
	Bandwidth Δf	U(1/16,1/8)
	Number of samples (N)	[500,1024]
BPSK	Carrier frequency $f_{c}$	U(1/8,1/4)
	Barker codes	{7,11,13}
	Number of code periods $N_{p}$	[100,300]
	Cycles per phase code (cpp)	[1,5]
Costas codes	Number change	[3,6]
	Fundamental frequency $f_{min}$	U(1/24)
	*N*	[512,1024]
Frank & P1 code	$f_{c}$	U(1/8,1/4)
	cpp	[1,5]
	*M*	[4,8]
P2 code	$f_{c}$	U(1/8,1/4)
	cpp	[1,5]
	*M*	2×[2,4]
P3 & P4 code	$f_{c}$	U(1/8,1/4)
	cpp	[1,5]
	*M*	2×[16,35]

**Table 3:** Confusion matrix for the system at signal-to-noise ratio (SNR) of −2 dB. The overall ratio of successful recognition (RSR) is 94.7%.

	LFM	BPSK	Costas	Frank	P1	P2	P3	P4
LFM	100	0	0	0	0	0	0	18
BPSK	0	100	0	0	0	0	0	0
Costas	0	0	100	1	0	0	0	0
Frank	0	0	0	91	3	0	4	0
P1	0	0	0	0	92	0	0	0
P2	0	0	0	0	0	100	0	0
P3	0	0	0	8	0	0	93	0
P4	0	0	0	0	5	0	3	82

**Table 4:** The testing environment.

Item	Model/Version
CPU	E5-1620V2 (Intel)
Memory	16 GB (DDR3@1600 MHz)
GPU	NVS315 (Quadro)
MATLAB	R2012a

**Table 5:** Computational complexity test (This paper/Lundén, Unit: Sec.).

	**LFM**	**BPSK**	**Costas**	**Frank**
−8 dB	55.604/86.324	51.332/82.374	54.875/84.279	56.336/87.022
−2 dB	54.979/86.111	51.195/81.560	54.009/84.183	56.294/86.654
10 dB	54.783/85.899	50.867/81.055	53.336/83.807	55.793/86.131
	**P1**	**P2**	**P3**	**P4**
−8 dB	58.628/88.282	56.754/88.360	58.830/87.798	54.895/85.999
−2 dB	58.422/87.923	55.801/88.180	58.107/87.353	54.428/85.187
10 dB	57.679/87.005	55.368/87.448	57.505/86.901	53.900/84.533

## Abbreviations

The following abbreviations are used in this manuscript:

LPI	Low Probability of Intercept
LFM	Linear Frequency Modulation
FM	Frequency Modulation
ENN	Elman Neural Network
BPSK	Binary Phase Shift Keying
FMCW	Frequency Modulated Continuous Wave
CWD	Choi–Williams Time-Frequency Distribution
PSK	Phase Shift Keying
FSK	Frequency Shift Keying
PCA	Principal Component Analysis
RSR	Ratio of Successful Recognition
SNR	Signal-to-Noise Ratio
EW	Electronic Warfare
STFT	Short-Time Fourier Transform
WVD	Wigner–Ville Distribution
PWD	Pseudo–Wigner Distribution
RD	Rihaczek Distribution
HT	Hough Transform
AWGN	Additive White Gaussian Noise
PSD	Power Spectral Density
